# Randomized clinical trial of pulpotomy using a premixed injectable calcium silicate cement on mature permanent teeth with reversible pulpitis

**DOI:** 10.1038/s41598-024-52818-6

**Published:** 2024-02-06

**Authors:** Sin-Yeon Cho, Seonghun Park, Yooseok Shin, Il-Young Jung

**Affiliations:** 1 Department of Conservative Dentistry, National Health Insurance Service Ilsan Hospital, Goyang, Gyeonggi-Do Korea; 2 Microscope Center, Department of Conservative Dentistry and Oral Science Research Center, College of Dentistry, Yonsei University, 50-1 Yonsei-ro, Sudaemun-gu, Seoul, 03722 Korea

**Keywords:** Health care, Health occupations

## Abstract

The aim of this two-center randomized controlled trial was to assess the outcomes and relative factors associated with pulpotomies performed using a premixed injectable calcium silicate cement, as compared to mineral trioxide aggregate in mature permanent premolar and molar teeth with reversible pulpitis. Included teeth were randomly divided into two groups according to pulpotomy material (ProRoot MTA [PMTA] group, Endocem MTA Premixed [EPM] group). After pulp exposure, the superficial pulp was either removed to a depth of 2 mm (partial pulpotomy) or completely amputated to the level of the root canal orifice (full pulpotomy). A 3-mm layer of either material was randomly placed over the pulp wound, followed by the application of a thin layer of a light-cured glass ionomer composite liner. The restoration procedure was then carried out during the same visit. After one year of treatment, the pulpotomy success rate was 94.4% (67/71), with no significant difference between the PMTA and EPM groups. The success rate was 93.9% in the PMTA group and 97.1% in the EPM group. There were no significant factors related to the procedures. EPM is a viable alternative to PMTA for single-visit pulpotomies of permanent premolars and molars.

## Introduction

When treating pulp exposure, root canal therapy (RCT) destroys the tooth structure through access opening and canal enlargement. In contrast, vital pulp therapy (VPT) avoids excessive loss of tooth structure and preserves the vital pulp, thereby maintaining the tooth's defensive properties^1^. Being a more conservative and predictable approach for permanent teeth with carious pulp exposure^2–4^, VPT (especially partial and full pulpotomy) is more beneficial than proceeding with RCT to preserve tooth structure and extend tooth longevity^5–8^.

Hydraulic calcium silicate cements, such as mineral trioxide aggregate (MTA), are recommended as the gold standard materials for VPT of exposed pulp because they have demonstrated reliable results in histological^9,10^ and clinical studies^11–15^. However, MTA has several disadvantages, such as coronal discoloration^16,17^, prolonged setting time^18^ and poor handling characteristics^19,20^, which hinder the completion of VPT and restoration during a single visit. In some clinical trials, a moist cotton pellet and an interim restoration were placed on unset MTA material, and the final restoration was placed at a subsequent visit^21–23^. In another clinical study, a moist cotton pellet was placed over the MTA for 45 min to achieve the optimal physical properties of MTA^24^, and the RMGI (Resin-Modified Glass Ionomer) base was adapted^25^ to complete the restoration procedure at the same visit^26^.

Calcium silicate cement products have been developed to reduce the setting time and improve handling. Biodentine (Septodont, Saint-Maur-des-fossés, France) is manufactured as a capsule containing material and liquid that is triturated by a mixer on demand. It sets within 6.5–45 min and is easy to handle, with a clay-like texture after mixing. Premixed products such as Endosequence BC RRM Fast Set Putty (EndoSequence; Brasseler, Savannah, GA, USA) and iRoot BP Plus (Innovative Bioceramix, Vancouver, Canada) are ready-to-use calcium silicate cements that are set for 20 min to 2 h after contact with a moist environment. Calcium-enriched mixture (CEM) cements have been also widely used because of their biocompatibility and adequate properties^27^. Theracal LC (ThLC; Bisco, Schaumberg, IL, USA) is a light-cured resin-modified calcium silicate liner for pulp capping that sets immediately upon light curing. Recent clinical studies have used these materials to perform VPT and restorative procedures in a single visit with favourable results^28,29^.

The recently developed Endocem MTA Premixed (EPM) (Maruchi, Wonju, Korea), a premixed injectable calcium silicate cement with a flowable consistency, contains zirconium dioxide as a radiopacifier instead of bismuth oxide and sets faster than ProRoot MTA (PMTA) (WMTA; Dentsply Tulsa, OK, USA)^30^. Its flowability allows direct delivery from the syringe to the target site. Despite its potential advantages, it has not been tested in clinical trials. Therefore, this study aimed to evaluate the clinical efficacy and outcomes of pulpotomy using EPM and compare them with pulpotomy using PMTA. The null hypothesis is that the outcome of pulpotomy with EPM does not differ from those of pulpotomy with PMTA.

## Methods

### Study design and population

This was a two-center randomized controlled trial of pulpotomy in permanent premolars and molars using PMTA and EPM. The appropriate independent ethics committees of Yonsei University Dental Hospital (YUDH) (IRB No. 2-2019-0038) and National Health Insurance Ilsan Hospital (NHIIH) (IRB No. NHIMC 2020-06-052-019) approved the study protocol, and all experiments were performed in accordance with relevant guidelines and regulations. The clinical trial was registered with the Clinical Research Information Service (Reg. No.: KCT0005734, 01/04/2021). We enrolled systematically healthy patients aged 11–82 years between January and October 2021.

Mature permanent premolar and molar teeth with reversible pulpitis were included: (1) no history of spontaneous pain; (2) a positive response to the cold test went away in less than 3–5 s; (3) negative responses to palpation and percussion; (4) a periapical index score ≤ 2 on the periapical radiograph^31^; (5) pulp exposure during caries removal or as a result of recent trauma; and (6) a healthy periodontium based on probing pocket depth ≤ 3 mm and mobility within normal limits. The exclusion criteria were as follows: (1) non-restorable crowns; (2) immature roots; (3) root resorption or pulp calcification; and (4) inability to control bleeding within 5 min after pulp exposure.

Patients who met the eligibility criteria were informed of the procedure, associated risks and benefits, and alternative treatment options. Written informed consent was obtained from all patients before enrolment.

### Hypothesis, sample-size determination and randomisation

All investigators and participants were blinded to the patient allocation. After this step, both (investigators and patients) were not blinded to materials due to the different consistency and manipulation. We postulated that pulpotomy using EPM would not be inferior to pulpotomy using PMTA. The average success rate of pulpotomy with calcium hydroxide is 20% lower than that of pulpotomy with hydraulic silicate cement, whereas the success rate of the control group is generally considered to be > 90%. Therefore, we set the non-inferiority margin at a success rate of 50–75% of − 20%, which is − 15–10%. Furthermore, 50 patients per treatment group were estimated to provide 80% power to detect a non-inferiority margin of − 0.12 with a one-sided α of 0.05, assuming a dropout rate of 20%.

**Figure 1:**
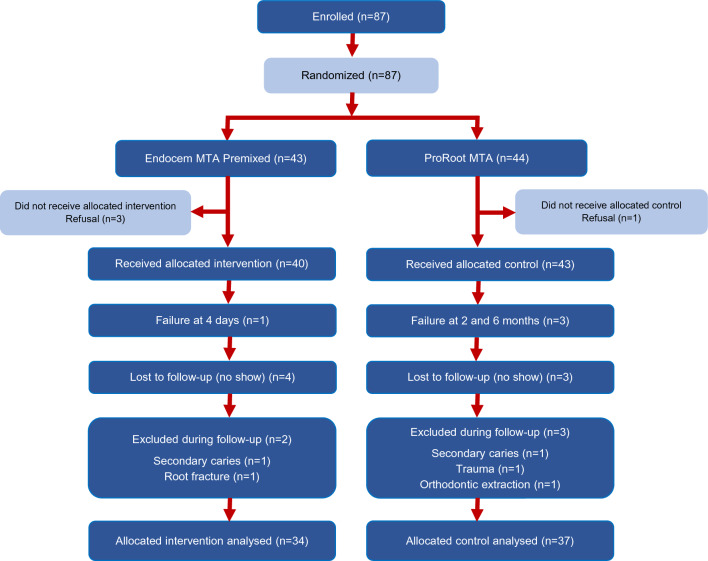
Flow chart of the study.

A clinical coordinator blinded to the study objectives generated a computer-generated list of random numbers using the Sealed Envelope website (https://www.sealedenvelope.com/) in phase 1 allocation using random block sizes of 6. The clinical coordinator assigned the affected permanent teeth to one of two groups: PMTA or EPM (Fig. 1).

### Treatment procedure

Six dentists, including five postgraduate residents and one faculty member from YUDH, one postgraduate resident, and one faculty member from NHIIH performed the procedures.

**Figure 2:**
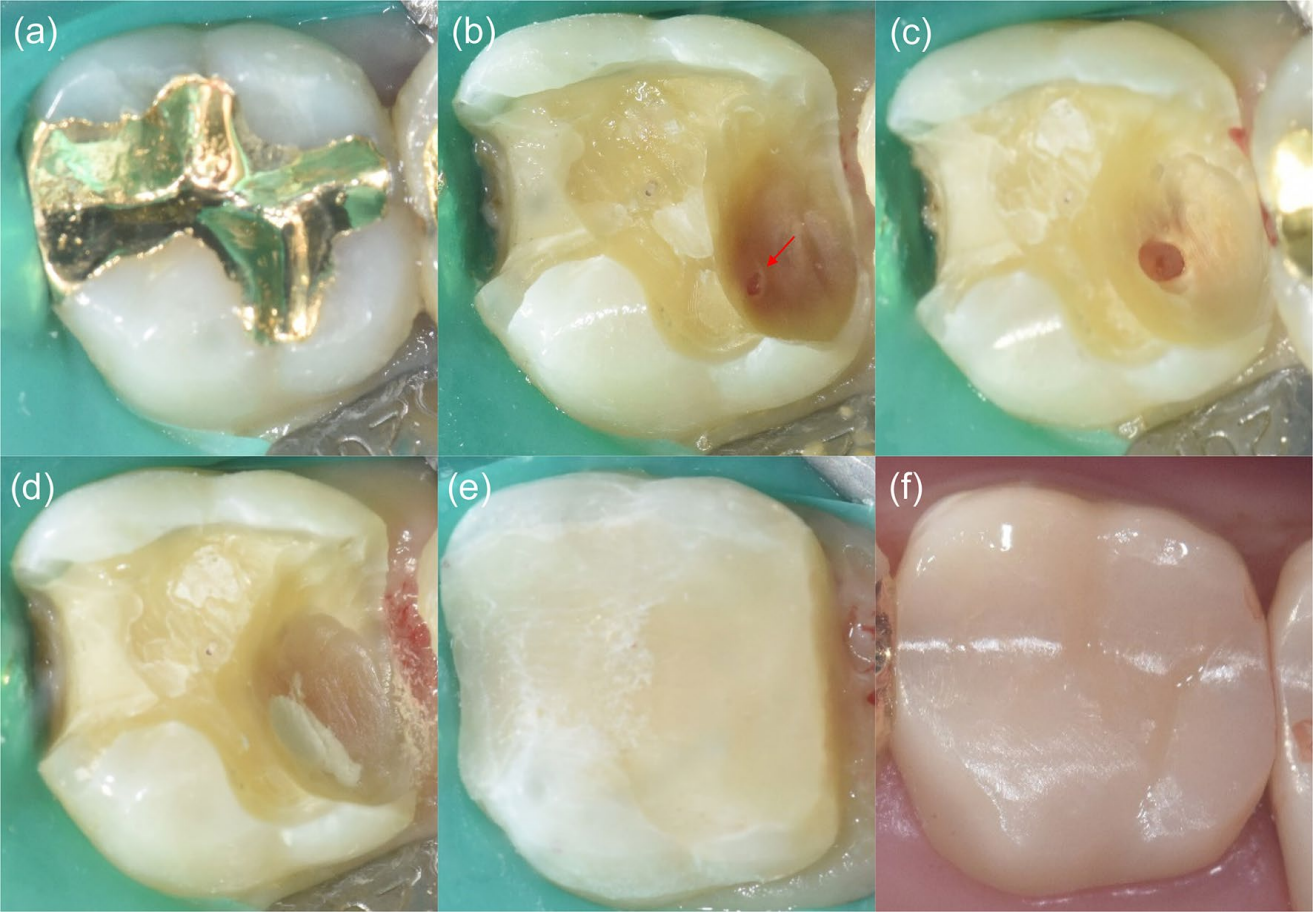
Clinical protocol for partial pulpotomy. (**a**) Preoperative clinical photograph. (**b**) Pulp exposure after non-selective caries removal (red arrow). (**c**) Hemostasis achieved after a few minutes by placing a cotton pellet soaked in 2.5% NaOCl against the exposed pulp tissue. (**d**) ProRoot MTA used to restore the exposed pulp. (**e**) Cavity restored with Ionoseal and composite resin restoration. (**f**) Final placement of the full zirconia crown.

**Figure 3:**
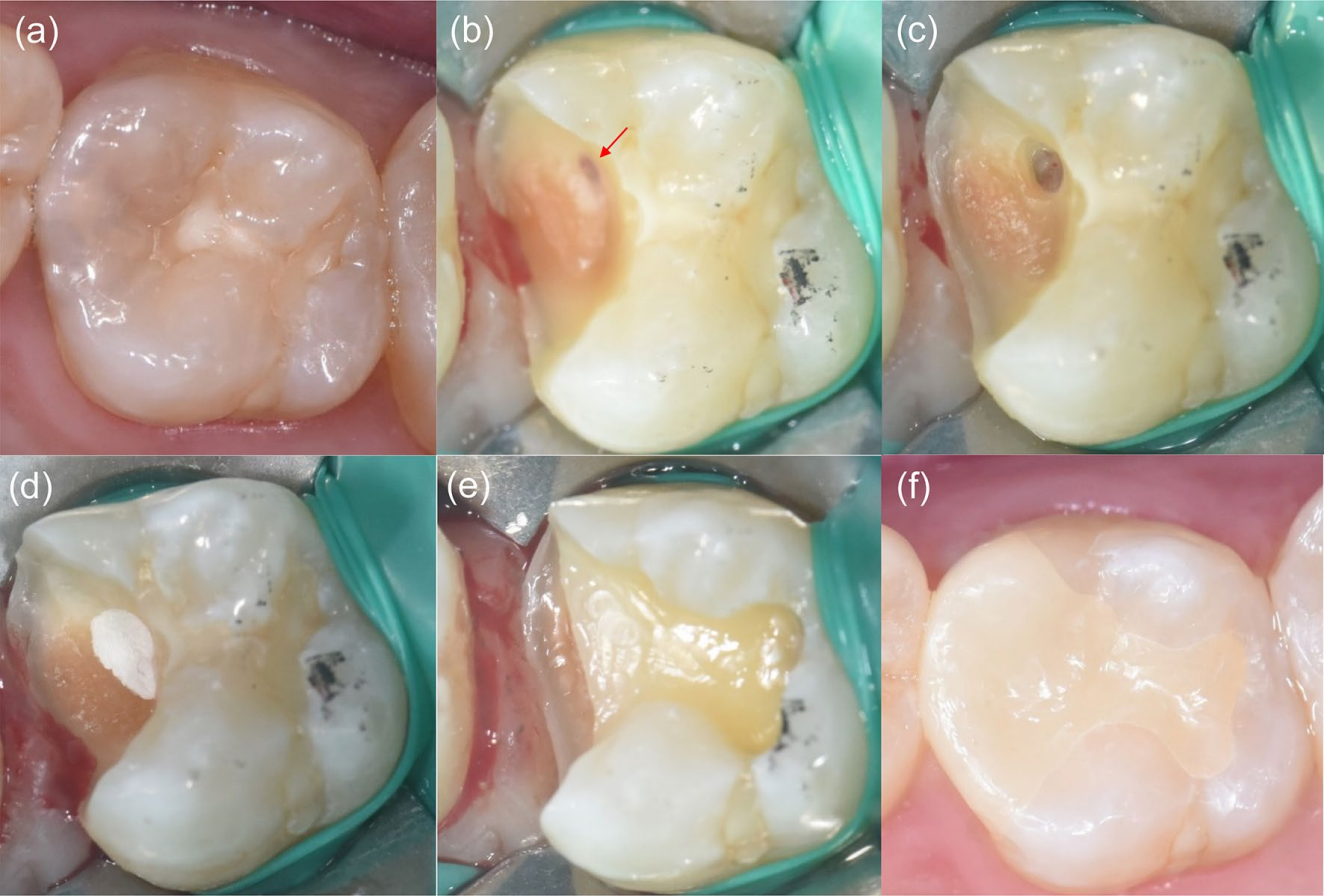
Clinical protocol for partial pulpotomy. (**a**) Preoperative clinical photograph. (**b**) Pulp exposure after non-selective caries removal (red arrow). (**c**) Hemostasis achieved after a few minutes by placing a cotton pellet soaked in 2.5% NaOCl against the exposed pulp tissue. (**d**) Endocem MTA used to restore the exposed pulp. (**e**) Cavity restored with Ionoseal and composite resin restoration. (**f**) Ceramic inlay placed.

Local anesthesia was induced using 1.8 mL of 2% lidocaine with 1:100,000 epinephrine (Huons, Sungnam, Korea), and caries removal was performed under rubber dam isolation (Figs. 2a,b, 3a,b). After pulp exposure, 2–3 mm of the pulp was removed to a depth of 2 mm using a sterile high-speed round-tapered diamond bur (Komet Dental, Lemgo, Germany) with copious irrigation (partial pulpotomy; Figs. 2c, 3c). If hemostasis was not achieved within 5 min, the pulp tissue was amputated to the level of the root canal orifice (full pulpotomy). The cavity was thoroughly irrigated using 2.5% NaOCl to remove superficial necrotic tissue for hemostasis^32^. After hemostasis was achieved, a randomly selected calcium silicate material was applied. In the PMTA group, the MTA was freshly mixed according to the manufacturer’s instructions and gently placed over the pulp wound and surrounding dentin to a thickness of 3 mm (Fig. 2d), whereas in the EPM group, the material was injected into the target area using a 19-gauge needle with a thickness of 3 mm (Fig. 3d). A moist cotton pellet was then placed to ensure the setting of both materials and removed after 5 min. A thin layer of light-cured glass ionomer composite liner (Ionoseal, VOCO, Germany) was applied and light-cured for 20 s. The cavity was restored using composite restoration at the same visit (Figs. 2e, 3e). If prosthetic restoration was required, it was started at the same or next visit (Figs. 2f, 3f). Immediate postoperative periapical radiographs were obtained.

### Postoperative evaluation

Participants were recalled at 3, 6, and 12 months after the pulpotomy, and radiographic and clinical examinations of the treated teeth were performed in each hospital. The all practitioners performed clinical examinations, but they did not only examine their own cases, but also randomly examined the cases of other practitioners. The clinical examinations included the presence or absence of pain (spontaneous or on percussion, biting, or palpation); swelling; sinus tracts; and other symptoms. Information about the materials was blinded to the examiners until the end of the study as it was not written in the medical record. Radiographic examinations included the detection of periapical radiolucency and evidence of pathological root resorption and calcific bridge formation, except in cases of crown restoration. Two operators who were not involved in this study and blinded to the type of MTA performed the radiographic analyses. They were given case numbers and radiographs to analyze, with no information about the patient or materials used. Where there was disagreement, the radiographs were re-evaluated until consensus was reached. Interexaminer reliability was determined with Cohen kappa statistics in accordance with Landis and Koch^33^. Pulpotomy success was determined based on the absence of clinical and radiographic signs and symptoms. Clearly visible calcific bridge formation on periapical radiographs was considered a successful calcific bridge formation, whereas its absence was considered a failure of calcific bridge formation.

### Statistical analysis

The chi-square test was used to assess the homogeneity of baseline characteristics between the two groups. The relationships between possible explanatory variables, treatment success, and calcific bridge formation were analyzed using logistic regression. Statistical analyses were performed using SPSS software version 29.0 (IBM, Armonk, NY, USA).

## Results

The interexaminer reliability for periapical radiolucency and calcific bridge formation was 0.869 and 0.629, indicating good and moderate agreement, respectively. A total of 87 teeth were enrolled, including 64 from the YUDH and 23 from the NHIIH. Four patients (four teeth) refused to participate after the allocated procedure, and 83 teeth were finally included. The four patients withdrew from the study regardless of the allocation because the allocated material was not explained to them. Five teeth, including two with secondary caries, one with trauma, one with a root fracture, and one with extraction due to orthodontic treatment, were excluded during follow-up. Seven patients were lost during the follow-up. Finally, 71 teeth, including 37 in the PMTA group and 34 in the EPM group, were clinically and radiographically evaluated at one year (91% recall rate) (Fig. 1). The chi-square test showed that the demographic and clinical variables were similarly distributed among the experimental groups (Table 1; *p* = 0.07–1.00).

**Table 1:** Baseline demographic and clinical features of all patients by treatment group.

	ProRoot	Endocem	Total	*P* value
Mean age	44.6 ± 21.2	39.9 ± 21.6	41.2 ± 22.4	1
Gender				
Female	22 (59.5%)	17 (50.0%)	39 (54.9%)	
Male	15 (40.5%)	17 (50.0%)	32 (45.1%)	1
Location				
Maxillar	23 (62.2%)	26 (76.5%)	49 (69.0%)	
Mandible	14 (37.8%)	8 (23.5%)	22 (31.0%)	0.31
Tooth				
Premolar	10 (27.0%)	8 (23.5%)	18 (25.4%)	
Molar	27 (73.0%)	26 (76.5%)	53 (74.6%)	0.72
Exposure				
Occlusal	8 (26.7%)	4 (16.7%)	12 (22.2%)	
Axial	22 (73.3%)	20 (83.3%)	42 (77.8%)	0.07
Type of pulpotomy				
Partial	30 (81.1%)	24 (70.6%)	54 (76.1%)	
Full	7 (18.9%)	10 (39.4%)	17 (23.9%)	1

ProRoot, ProRoot MTA; Endocem, Endocem MTA Premixed.

Four clinical failures were noted within six months of treatment (Table 2). At 1 year follow-up, no patients showed clinical or radiographic signs or symptoms of failure. Therefore, the overall pulpotomy success rate was 94.4% (67/71); 93.9% in the PMTA and 97.1% in EPM with no significant difference between the groups. Full and partial pulpotomies were performed in 17 (overall success rate 94.1%) and 54 teeth (overall success rate 94.4%), respectively. The site of exposure was analyzed only in cases of partial pulpotomy. The success rates for the occlusal and axial exposures were 91.7% and 95.2%, respectively. No significant variables were associated with treatment success (Table 3).

**Table 2:** Characteristics of the failure cases during the follow-up period.

Age	Tooth type	Exposure type	Exposure site	Material	Time	Reason of Failure
30	Molar	Caries	Occlusal	ProRoot	2 Months	Spontaneous pain
19	Premolar	Caries	Axial	ProRoot	6 Months	Spontaneous pain
62	Premolar	Caries	Full pulpotomy	Endocem	4 Days	Spontaneous pain
54	Molar	Caries	Axial	ProRoot	6 Months	Spontaneous pain

ProRoot, ProRoot MTA; Endocem, Endocem MTA Premixed.

**Table 3:** Characteristics of included patients and bivariate associations with outcomes (n = 71).

Variables	Total N = 71		Treatment success					Calcific bridge formation				
			Success		Failure		*P* value	Success		Failure		*P* value
	N	%	N	%	N	%		N	%	N	%	
Age							0.32					0.33
< 21	15		14	93.3	1	6.7		5	55.6	4	44.4	
21–40	19		18	94.7	1	5.3		6	33.3	12	66.7	
41–60	19		18	94.7	1	5.3		3	21.4	11	78.6	
> 60	18		17	94.1	1	5.9		2	22.2	7	77.8	
Gender							0.56					1.00
Male	32		31	96.9	1	3.1		6	31.6	13	68.4	
Female	39		36	92.3	3	7.7		10	32.3	21	67.7	
Tooth type							0.28					0.73
Premolar	18		16	88.9	2	11.1		5	38.5	8	61.5	
Molar	53		51	96.2	2	3.8		11	29.7	26	70.3	
Jaw							0.70					0.19
Maxilla	22		21	95.5	1	4.5		9	25.7	26	74.3	
Mandible	49		46	93.9	3	6.1		7	46.7	8	53.3	
Material							0.17					0.36
ProRoot	37		34	93.9	3	6.1		6	24.0	19	76.0	
Endocem	34		33	97.1	1	2.9		10	40.0	15	60.0	
Exposure site							0.67					0.14
Occlusal	12		11	91.7	1	8.3		6	54.5	5	45.5	
Axial	42		40	95.2	2	4.8		10	25.6	29	74.4	
Pulpotomy type							0.98					
Full	17		16	94.1	1	5.9						
Partial	54		51	94.4	3	5.6						

ProRoot, ProRoot MTA; Endocem, Endocem MTA Premixed.

**Figure 4:**
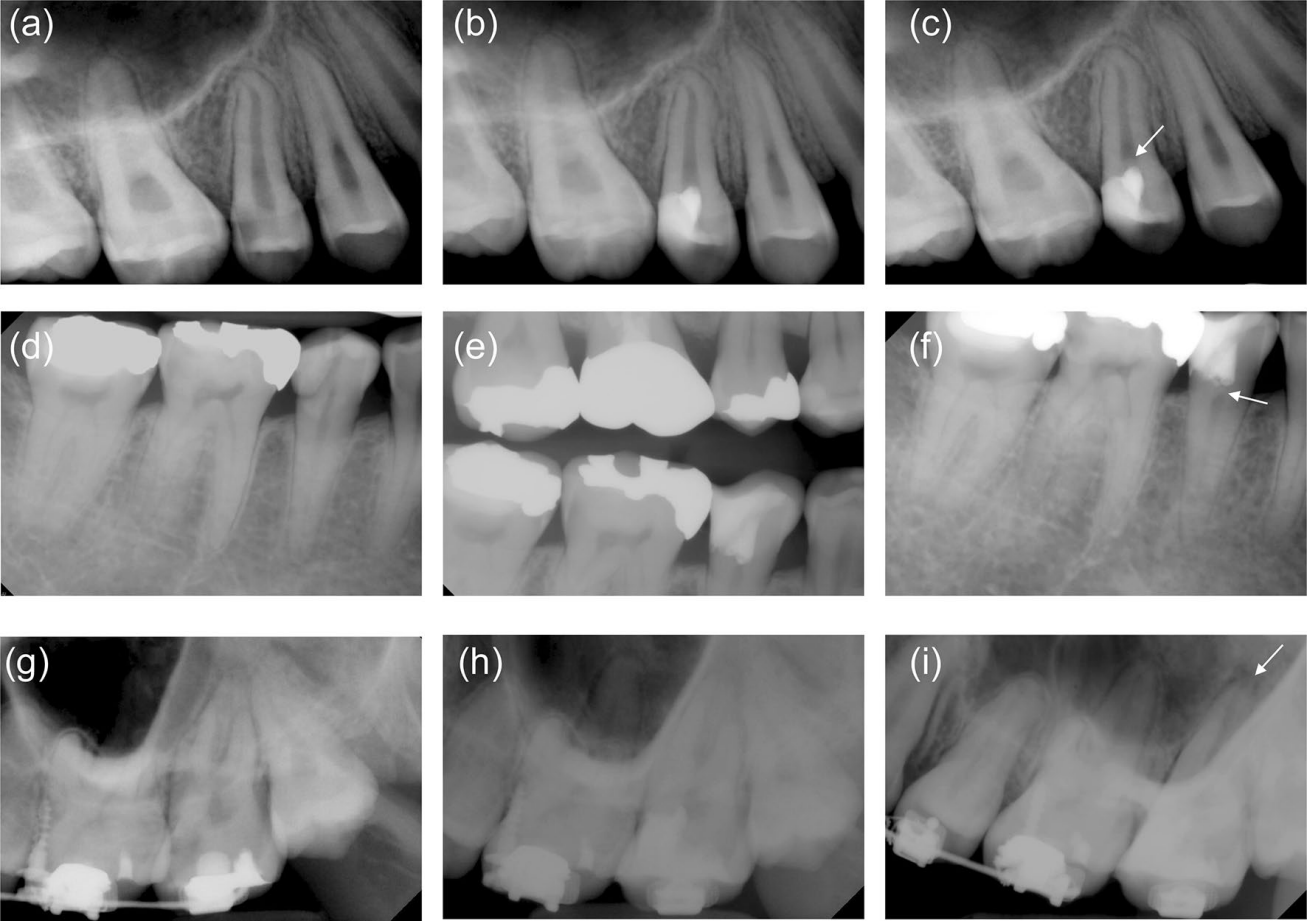
Outcome of teeth treated with partial pulpotomy. (**a**) Preoperative radiograph of tooth 15 with extensive caries. (**b**) Immediately postoperative radiographs after partial pulpotomy using Endocem MTA Premixed. (**c**) 12-month postoperative radiographs showing calcific bridge formation (white arrow) and normal periapical tissue. (**d**) Preoperative radiograph of tooth 45 showing caries close to the pulp. (**e**) Immediately postoperative radiographs after partial pulpotomy with ProRoot MTA. (**f**) 12-month postoperative radiograph showing calcific bridge formation (white arrow) and normal periapical tissue. (**g**) Preoperative radiograph of tooth 27 with extensive caries. (**h**) Immediately postoperative radiographs after partial pulpotomy with ProRoot MTA. (**i**) 2-month postoperative radiograph showing the periapical lesion around the palatal root. The patient complained of severe pain upon cold stimulus.

Owing to extracoronal restoration, it was not possible to assess calcific bridge formation in four cases, including two in the PMTA group and two in the EPM group. Calcific bridge formation occurred in 6 (24.0%) cases of 25 partial pulpotomy cases in the PMTA group and 10 (40.0%) cases of 25 partial pulpotomy cases in the EPM group (Table 3, Fig. 4). The odds of calcified bridge formation were approximately two times higher in the under-21 age, mandibular teeth, and occlusal exposure groups than in other groups. None of the independent variables significantly affected calcific bridge formation after partial pulpotomy (Table 3).

## Discussion

This study aimed to evaluate the clinical efficacy and outcome of pulpotomy using a premixed injectable calcium silicate cement with a flowable consistency, EPM, and compare it with pulpotomy using PMTA. The outcome of pulpotomy with EPM was not significantly different with those of pulpotomy with PMTA, therefore, the null hypothesis was not rejected. This study provides insights into the clinical efficacy and handling of this new type of calcium silicate cement. If proven as effective as PMTA, EPM could provide a more convenient and efficient alternative for VPT and restorative procedures. Overall, our study contributes to the ongoing efforts to improve and refine VPT techniques and materials for managing exposed pulp.

In the present study, partial pulpotomy was performed during a single visit. After applying the MTA cement, the cement was covered with RMGI to continue the restorative procedure. This is different from the traditional method of placing a wet cotton pellet over the MTA and temporary restoration to ensure the complete setting of the MTA cement^21–23^. One-sided hydration of the pulp is sufficient for the MTA setting^34^, and glass ionomer cement restoration over MTA does not affect the MTA setting^24^. Therefore, recent studies on single-visit pulpotomies have placed glass ionomer cement over MTA cement^26,28,29^.

The study reported an overall success rate of 94.4%, which is considered excellent for pulpotomies. The success rate was assessed based on clinical and radiographic criteria, and the results were evaluated at six months and one year after treatment, as recommended in the position statement^35^. Although a 1-year observation period may be limited in terms of the reliability of a clinical trial, it is sufficient to assess the validity of the materials used as in our study, all failures occurred within 6 months (Table 2). The success rate in this study was higher than that reported in recent pulpotomy studies using traditional MTA and newer calcium silicate cements over the same observation period^23,26,28,29,36,37^. This may be because the recent pulpotomy studies included teeth with irreversible pulpitis, whereas this study included teeth with normal pulp or reversible pulpitis. Therefore, a direct comparison is not appropriate; however, we believe that the results of this study are sufficient to demonstrate the feasibility of the new material for pulpotomy.

This study found no statistically significant differences in success rates between the two materials used; MTA and calcium silicate cement showed similar clinical and radiographic results. This suggests that calcium silicate cement with a flowable consistency can be used as an alternative to MTA for pulpotomy procedures with comparable efficacy. Although EPM has a faster initial setting time than PMTA, and Palma et al.^38^ suggested that all of the calcium silicate-based cements tested allow restorative procedures to be performed after initial setting, allowing the treatment to be completed in a single appointment, the effects of etching and bonding agents on EPM have not yet been verified. Therefore, the protocol for the application of RMGI over the cements was performed in our study, similar to other single-visit studies^26,28,29^.

The success rate of a pulpotomy is influenced by several factors, including the extent of pulpitis, tooth maturity, and restoration quality after the procedure; these factors should be considered when selecting the material and technique for pulpotomy to ensure the best possible outcome. In our study, we analysed several factors, including age, sex, tooth type for overall pulpotomy and exposure site for partial pulpotomy; however, no significant factors were identified (Table 3). Other recent pulpotomy studies have failed to identify significant factors^23,26,28,29,37,39^. Even the exposure site, which was a significant factor in a previous direct pulp-capping study^12^, was not a significant factor in recent partial pulpotomy studies^26,40^. This is likely due to the improved biocompatibility and sealing ability of the materials. The cavity-forming nature of the pulpotomy procedure resulted in accurate filling of the materials, reducing the influence of factors other than the pulp condition on the procedure.

A calcific bridge is a structure formed within the dental pulp tissue in response to injury or trauma and acts as a barrier between the healthy pulp tissue and the external environment, protecting the pulp tissue from further injury or infection. In this study, calcified bridges were found in 32% (16/50) of the teeth with a partial pulpotomy, which is higher than in other studies that have evaluated calcified bridges^23,29^; however, we were unable to determine the reason for these differences, as each study did not provide their evaluation criteria for calcified bridges. Calcific bridges were found more frequently in younger patients with EPM rather than PMTA and with occlusal exposure rather than axial exposure, although these were not statistically significant.

The teeth included in this study had pulp exposure either during caries removal or as a result of recent trauma. In addition, there was no history of spontaneous pain and lingering pain to cold test. Thus, the preoperative condition of the pulp was diagnosed as reversible pulpitis. Recently, Santos et al.^41^ suggested that the radiographic and histologic results of full pulpotomy are not compromised by short-term preoperative pulp inflammation. This suggests that full pulpotomy may be effective for teeth with irreversible pulpitis. Further research is needed on this issue.

Several limitations should be considered when interpreting the results of this study. First, eight practitioners participated in this study, and differences in skills may have influenced the results. All eight practitioners are from the same hospital and have the same concept of the procedure. Before starting the study, all the practitioners had a meeting to standardize the diagnostic criteria and procedures. Nevertheless, the participation of multiple practitioners is a limitation of this study. Second, the observation period in this study was only one year, which is not long enough to observe bacterial microleakage or long-term pulpal response. Third, this study did not fulfill the required sample size because of the limited time for patient recruitment. Fourth, periapical radiographs were used to assess the calcific bridge formation, although bitewing radiography and cone-beam computed tomography were more accurate. Fifth, when the hemostasis was not achieved within 5 min in this study, we performed the full pulpotomy instead of partial pulpotomy. Although Careddu and Duncan argued that bleeding time is not a significant factor in treatment success, we performed full pulpotomy for the convenience of the procedure rather than focusing on the success rate.

## Conclusion

This study provides valuable insights into the use of premixed injectable calcium silicate cement with a flowable consistency as a potential alternative to MTA cement for pulpotomies.

## Data Availability

The data included in this study are available from the corresponding author upon reasonable request.
